# Challenges in day-to-day midwifery practice; a qualitative study from a regional referral hospital in Dar es Salaam, Tanzania

**DOI:** 10.1080/16549716.2018.1453333

**Published:** 2018-04-06

**Authors:** Hanna Strømholt Bremnes, Åsil Kjøl Wiig, Muzdalifat Abeid, Elisabeth Darj

**Affiliations:** a Department of Public Health and Nursing, NTNU, Norwegian University of Science and Technology, Trondheim, Norway; b Department of Obstetrics and Gynecology, Temeke Regional Referral Hospital, Dar es Salaam, Tanzania; c Department of Obstetrics and Gynecology, St Olavs Hospital, Trondheim, Norway; d Department of Women’s and Children’s Health, Uppsala University, Uppsala, Sweden

**Keywords:** Health workers’ perceptions, heavy work, shortage of staff, demoralization, African context

## Abstract

**Background**: Maternal and infant mortality rates in Tanzania have decreased over the past decades, but remain high. One of the challenges the country faces, is the lack of skilled health care workers. High fertility rates make midwives and their patients particularly susceptible to stress as a result of understaffing.

**Objective**: This paper explores the challenges midwives face in their day-to-day practice at a regional referral hospital in Tanzania, and investigates which measures the midwives themselves find necessary to implement to improve their situation.

**Methods**: A qualitative study design with focus group discussions (FGDs) was employed to explore which challenges the midwives experienced. Each focus group consisted of five to six midwives. A FGD topic guide covering challenges, consequences, motivation, ideal situation and possible solutions was used. These data were analyzed using Systematic Text Condensation.

**Results**: A total of 28 Midwives, six men and 22 women, participated in five FGDs. Four categories emerged from the collected material: Feelings of demoralization, shortage of resources, societal challenges and personal struggles. A feeling of demoralization was especially prevalent and was caused by a lack of support from the leaders and little appreciation from the patients. Shortage of resources, and shortage of personnel in particular, was also highlighted as it led to an excessive workload resulting in difficulties with providing adequate care. These difficulties were intensified by lack of equipment, facilities and a non-optimal organization of the healthcare system.

**Conclusion**: The challenges revealed during the FGDs prevent the midwives from providing sufficient midwifery care. To improve the situation, measures such as supportive leadership, reduction of workload, increasing availability of equipment and increasing knowledge of reproductive health in society, should be taken.

## Background

Maternal and neonatal health have been one of WHO’s key priorities for decades. The Millennium Development Goals (MDGs), and the Sustainable Development Goals (SDGs), have especially targeted the maternal mortality ratio (MMR) and the infant mortality ratio (IMR) [1]. In Tanzania, working to reach the MDGs has been important [2]. Tanzania has a total population of 45 million people, where women of childbearing age make up 47.1% [3]. The Total Fertility Rate is 5.2 [4]. The infant mortality rate is 43 per 1000 live births, which is lower than the average infant mortality in sub-Saharan region of 79.1 per 1000 [4,5]. The maternal mortality ratio (MMR) was estimated to be 556 per 100.000 births in 2016. This is higher than the ratios reported in 2010, but a decrease from the 2005-levels of 578 per 100.000 [4,6].

**Table 1.** Method of Systematic Text Condensation for data analysis using an example from the collected material.

Theme	Meaning unit	Category	Subcategory
General shortage	‘There are so many patients, but the staff is few’	Shortage of resources	Personnel

**Table 2.** Overview of main categories and subcategories emerging from the focus group discussions with the midwives.

Categories	Sub-categories
Feelings of demoralization	Blamed by patients
	Lack of support from superiors
Shortage of resources	Personnel
	Equipment
	Facilities
Societal challenges	Low level of education in the population
	Lack of collaboration within the health system
Personal struggles	Health problems
	Lack of personal development
	Family life troubles

The fluctuations in MMR, and persistent high levels of mortality in both mothers and children, illustrates the required need for further research on how to improve the situation, which does not yet meet the goals postulated by WHO. One of the main challenges is that there are only four nurse-midwives per 10.000 inhabitants [7], which is considerably lower than the minimum of 23 per 10.000 recommended by the WHO [8]. This shortage of qualified personnel affects both healthcare professionals and their patients. The high fertility rate and large number of fertile women amplifies this problem and makes the shortage of midwives especially challenging.

Midwives and the barriers they face have been studied before and are regarded as key for improving child and maternal health. A worldwide systematic review on midwifery care in low and middle-income countries argues that barriers for providing good midwifery care are created by social, economic and professional factors [9]. The consequences of these barriers are feelings of exhaustion and moral distress, which in turn affects the quality of the care provided [9]. A study from Tanzania (2015) identified three main barriers to providing quality midwifery care in the country; poor-working conditions, lack of status and perceived lack of knowledge [10].

The aim of this study was to explore and highlight the challenges the midwives face in their day-to-day practice, and to investigate which measures the midwives find necessary to implement to improve their condition. This may enable actions to be taken informed by the midwives’ firsthand experience of the situation. Seeing their own suggestions being taken into account may lead to increased motivation, thereby improving the midwives’ working conditions, which in turn could better maternal and child health in the country.

## Methods

### Study design

A qualitative study design using focus group discussions (FGDs) was employed to explore the challenges midwifes at a regional referral hospital in Dar es Salaam face in their day-to-day practice. FGDs was considered a suitable method for data collection as it allows interactions between participants and provides the possibility of obtaining multiple views and perceptions of a subject [11]. The COREQ-guidelines for reporting qualitative research were followed [12].

### Study setting

This study was conducted at the Obstetrics and Gynecology (OBGYN) department at a Regional Referral Hospital in Dar es Salaam, Tanzania. Tanzania has a pyramidal healthcare system organized in five levels, where regional referral hospitals serve as the fourth level of healthcare facilities. The hospital in question is responsible for providing healthcare to a population of around 1.6 million people in one district of Dar es Salaam [3]. The facility gets both self-referrals and referrals from the 55 government owned district health care facilities in the surrounding region [5]. The OBGYN department at the hospital has five wards; Antenatal ward with a capacity of 12 beds, labor ward with 20 beds, post-operational ward with 10 beds, post-natal ward with 30 beds and intensive care unit (ICU) with 6 beds. The hospital has approximately 17,000 annual deliveries, between 40 and 50 each day. The staff consists of doctors and nurse-midwives. Most deliveries and other routine work are performed by nurse-midwives. There are two to three midwives present per shift in the Labor ward, making the patient-nurse-ratio 10:1. In the other wards, except the ICU, there is fewer staff, with one midwife being responsible for up to 30 patients during afternoons and nights. When a complication arises a specialist doctor on call is summoned for intervention.

### Study participants

Participants were recruited from the OBGYN department at the Regional Referral Hospital using a purposive sampling technique [13]. Midwives of both genders, all ages and of varying seniority were included in order to ensure a rich and diverse data material. The only inclusion criterion was to be employed as a midwife at the hospital and to be willing to participate in the study. Thirty nurse-midwives were asked to take part in the FDGs, of which two declined. In total, 28 participated, among them six men and 22 women. Their age ranged from 23 to 57, and their working experience from 2 months to 33 years.

### Data collection

Five FGDs were conducted over a 2 week period with 5–6 midwives in each group. The group size was decided based on recommendation from Malterud [11], who regards 5–6 participants as suitable number to secure both participation and presentation of multiple views. After the 5 FGDs were conducted, the material was perceived to be saturated as no new topics emerged during the final interview [11]. To avoid compromising the patient care, the interviews took place during the least busy time of day, after ward rounds and before changing of shifts, and with midwives from different wards. The interviews were conducted in Kiswahili. An independent translator, who works as a midwife at another hospital in Dar es Salaam, moderated the FGDs assisted by the two first authors who observed and took notes. An FGD guide with topics covering specific areas was used, such as: challenges, consequences of the challenges, motivation, ideal situation and possible solutions. The FGDs were on average 60 minutes long and were audio-recorded with permission from the participants. The audio-recordings were translated verbally into English by the translator after the FGDs and simultaneously transcribed by the first authors into written English transcripts. The written translations were verified against the audio-recordings for accuracy by the local supervisor. No significant incongruences or mismatches were found.

### Data analysis

The qualitative method of Systematic Text Condensation (STC), as described by Malterud [14], was applied to the material. This proved to be a suitable method for analyzing the manifest content of the material and provide a systematic presentation of the midwives’ experiences and situation. The main steps of the analysis involved interpretation of data through multiple readings of the transcripts and identification of themes and meaning units. The meaning units were then coded and grouped into categories and subcategories which were labeled at a manifest level and validated against the original transcripts (Table 1).

## Results

All participants actively participated in the discussions. The atmosphere in the interview room was characterized by a shared frustration. The topics of challenges and solutions sparked passionate debates and the midwives eagerly shared their experiences. Four key categories emerged from the collected material: feelings of demoralization, shortage of resources, societal challenges and personal struggles (Table 2).

### Feelings of demoralization

One of the main concerns reported by the participants during the interviews was a feeling of demoralization induced by both their clients and their supervisors.

#### Blamed by patients

The midwives felt that when something went wrong, e.g. maternal death or stillbirths, the patients and their relatives would always blame them.

*‘*I don’t think there is any nurse who would be happy for anybody to lose their baby, or that there is any nurse who would want a woman to die. These things are accidents, but the patient will always blame the nurse’. (Midwife 4 FGD3)

They reported to have been verbally abused by their patients, something that made them feel that their hard work was being undermined. It was their general impression that midwives had a bad reputation in the society, and they wished that the population would learn more about the work midwives do. Their hope was that this would make patients and their close ones appreciate their work to a greater extent.

#### Lack of support from superiors

The midwives also experienced a loss of motivation at work due to limited support from their superiors, something that contributed to their feeling of demoralization. They felt that they had no advocate in their leaders, and that the leaders always were on the patient’s side in conflicts. They wanted an arena for dialogue with their superiors and the possibility to defend themselves in situations where complications had occurred.

‘Well, our leaders are on the side of the patients, not us. That’s just politics. It’s painful, we always get the punch’. (Midwife 2 FGD1)

*‘*Yes, that’s right. And you’ll just have to forget that something unfair happened to you yesterday’. (Midwife 5 FGD1)

The midwives also found it problematic that they were not compensated for working overtime, and this contributed to them feeling even more devalued in their work. Lack of other incentives for working, like tea or compensation for transport when staying late, enhanced this feeling.

### Shortage of resources

The participants described a shortage of personnel, equipment and facilities that leads to problems with overwork and risk of infections for the midwives, and poor monitoring, delays in treatment and unnecessary complications for the patients.

#### Staff

All the participating midwives reported a substantially higher patient-nurse-ratio than the one recommended at the hospital. Two midwives could be responsible for up to 60 women in different stages of labor per shift when delivery frequency is at its highest. To make the situation bearable, each midwife has to work longer hours and more shifts than they are supposed to. ‘You come to work in the morning, and no matter how tired you are you can’t leave work when you are supposed to because there are too many women who needs your help.’ (Midwife 3 FGD1)

All participants reported being tired, something that affects patient care. Several of the midwives confessed that they sometimes acted rudely towards patients because they were tired and impatient. Increasing the amount of staff was seen as one of the most important factors for improving the working conditions and the care provided to patients.

‘Everybody is busy! Let’s talk about reality. So there are two midwives and there are 60 women in different stages of labor. And all these women expect to deliver under supervision of a midwife and afterwards have their babies assessed, is that possible? It’s impossible!’ (Midwife 5 FGD3)

#### Equipment

The Tanzanian government state that delivery services should be free for all women, but the participants reported that the government does not provide the hospital with enough equipment to cover their most basic needs. They lacked everything from essential supplies like gloves, masks, syringes and catheters, to more advanced material like digital monitors of blood pressure and fetal heart rate. The lack of equipment endangers both the midwives and their patients.

‘We’ve got too low amounts of equipment compared to the number of women in the ward. Due to this we have to ask them to buy their own equipment, which makes the women angry because they’ve heard in the media that hospital treatment is free’. (Midwife 1 FGD1)

The midwives reported a constant fear of getting infected with for example hepatitis, HIV or TB because of the lack of protective gear, something that made them more reluctant to help women with known infections. They were convinced that if they had better and more available equipment, they would feel safer. More advanced equipment would also make it possible for them to monitor and follow-up patients more adequately and make it easier to determine which patients need their help the most.

‘The protective gloves are seasonal. It’s uncommon to help a woman deliver without getting blood on your forearms.’ (Midwife 5 FGD3)

When the hospital is out of equipment, the midwives have to ask their patients to go buy the equipment to be able to provide the required care. Since policy states that delivery services are free, the midwives reported that asking for equipment was often interpreted by the clients as asking for money for the midwives’ own personal use.

#### Facilities

The participants reported a shortage of necessary facilities needed to provide good obstetric care. The lack of available theaters for conducting C-sections was the prime concern. The hospital only has one operating theater, which is shared by all departments. A frequently reported problem was the need to bring a patient in for an emergency C-section, but having to wait for several hours because the theater was busy, resulting in stillbirths that could otherwise have been avoided.

‘I had an incident where I took two women to the theatre and they had to wait in line. One got a low score baby and the other one was a stillbirth.’ (Midwife 2 FGD1)

Another issue was that there was only one ambulance available for the entire hospital. If the theater was busy and a woman needed a C-section, it would, in theory, be possible to refer her to the national hospital, but the lack of ambulances makes this difficult.

### Societal challenges

During the FGDs it became clear that there were several societal factors that made the working situation at the hospital difficult. The midwives found the low education level in the population especially challenging. They thought that insufficient education caused delayed arrivals at the hospital. Lack of cooperation between health care facilities was another problem, especially the lack of a well-functioning referral system.

#### Low level of education in the population

The hospital is located in one of the most densely populated areas of Dar es Salaam. The participants reported that the population in the area has a low level of education, which affects their knowledge of family planning and reproductive health. The nurse-midwives postulated that the lack of knowledge resulted in more complications, because women arrived late at the hospital, often only if complications arose. A factor that contributed to an increase in complications was the use of local herbs to speed up labor, a common practice in the area, according to the participants. They wished that they had the resources to help educate women on reproductive health. This would lead to less complications and unnecessary deaths.

‘The clients that we take care of are challenging in themselves. These women come in when they have used local herbs to speed up the labor, or they delay coming to the hospital. Some come when it’s almost pushing time or when they have macerated babies’. (Midwife 3, FGD2)

#### Lack of cooperation within the health system

The healthcare system in Tanzania has a pyramidal organization where the women are supposed to attend antenatal check-ups in their local clinics during pregnancy. This was problematized by the midwives because some of the peripheral clinics did not collect or transfer vital information about the women, for example HIV-status, measuring of HB-levels and blood pressure (BP) control. This is problematic in emergencies and may cause delays in treatment.

‘BP is very crucial for a pregnant woman. If you don’t measure these women and they have no idea what eclampsia is, that’s when they end up coming here with eclampsia’. (Midwife 5 FGD4)

Another challenge the midwives pointed out was the fact that the women come without a referral letter, which increases the patient load. The hospital has many normal deliveries that could have been taken care of at a lower healthcare level. This would have eased the workload and given the midwives more time to care for the remaining patients.

Furthermore, the cooperation with the doctors was mentioned as a problem. The participants reported that they felt underrated by the doctors and that this sometimes led to delays in diagnosis and treatment because the doctors did not trust the midwives’ observations. Defined routines and teamwork between doctors and nurses was mentioned as a possible way of improving the healthcare provided.

### Personal struggles

The participants reported that the heavy workload and stressful situation at work affected their personal and family lives in several ways. They experienced both physical and mental health problems, limited personal development and trouble with their families.

#### Health problems

The long shifts and heavy workload affect the midwives both psychologically and physically. They reported that the heavy lifting and the long hours causes back aches and disc prolapses. Some also had miscarriages. Because the work is so stressful, the midwives seldom have time to eat or sit down to rest, and they come home exhausted. Many find it difficult to leave work behind when they return home and continue thinking about their patients after their shift has ended.

‘We get health problems and severe back aches due to disc prolapse. A lot of the midwives have disc prolapse’. (Midwife 2, FGD2)

#### Lack of personal development

Opportunities for further education and promotions are limited, and the midwives felt that this makes it harder for them to gather motivation for their work. Low salaries and restricted compensation for overtime work contributed to this demotivation. The midwives reported that the few trainings and update courses that actually are arranged are unavailable to them, either because they are too busy working in the wards or because the people who work in administration are prioritized. More access to trainings and possibilities for career advancement would motivate them more and make them able to perform better at work.

#### Family life troubles

The participants highlighted that the long shifts at work are difficult to combine with family and social life. They complained that they do not see their spouses and children enough. They have no time for household chores or to follow up on their children’s school work. They were concerned that their maids were raising their children, and it bothered them not to be in control of their upbringing themselves.

‘Well your family perishes; you’re making other people’s family happy, but you’re making your own sad. You work on somebody else’s happiness when you’re killing your own back home’. (Midwife 2, FGD1)

## Discussion

The most prevalent findings in this study was the feeling of demoralization. Other factors of importance were personal struggles, shortage of staff, equipment availability, and unawareness and challenges in society. The feeling of demoralization and lack of motivation is in line with findings from other studies conducted in the region [15–17]. Positive support from supervisors have been demonstrated to be of importance for the quality of services that health workers are able to deliver [18]. In the World Health report on improving performance in healthcare, the WHO stress that supportive supervision can contribute to improved performance of health workers [18,19]. In situations where employees experience lack of motivation, consequences are lack of courtesy to patients, poor process quality and failure to treat patients at an appropriate time [20]. The health outcomes of patients are therefore critically dependent on the nurses’ motivation [21]. Changing the management strategy, or providing supportive management training for supervisors, are documented measures that can be taken to increase the level of motivation in the workplace [16,18]. Another important factor to improve performance is adequate salaries [21]. Hospitals where at least a minimum of allowances are paid, tend to have a more motivated work force, and consequently more content patients, according to Tibandebage et al. [16].

The experienced lack of opportunities for career advancement and personal development, which were presented in the category ‘personal struggles’, also contribute to the feeling of demotivation. Continued education is one of the most effective ways to heighten midwives’ motivation and cultivate midwives’ skills [22]. Skilled and motivated midwives with possibilities for career development has proven to be an efficient way to reduce mother and child mortality [5]. Another way to heighten motivation is through promotion. Providing midwives with the possibility of future education, and/or possibilities for promotion is therefore something that may lead to higher staff retention and a more motivated staff.

Shortage of staff, equipment and facilities were other reported barriers to providing adequate midwifery care, a finding supported by other studies from the region [16,17]. Delivery attended by skilled personnel with appropriate supplies and equipment has been found to be strongly associated with reduction of child and maternal mortality [23,24]. Taking measures to increase access to human resources is of great importance to improving patient care. One option is to bring in more qualified staff, but a severe shortage of healthcare providers in the region makes this challenging [23]. Another way of reducing the workload suggested by the midwives is to make changes to the organization of the healthcare service. If the referral system is more controlled and the patients are required to have a referral letter from their district hospital before they can come to the referral facility, the workload may be reduced. Shortage of equipment is ideally solved by getting more resources. However, governmental spending on health in percentage of GDP is decreasing, making less resources available [25]. At the same time, the government proclaim that maternal healthcare during pregnancy and delivery is free [26]. This is a challenge for the midwives, since it causes a gap between government policy and reality. The goal should be to provide free healthcare, as this has been shown to reduce mortality [26], but a prerequisite for this is an increase in governmental spending on health. Other factors important for the reduction of mortality are advanced monitoring equipment and available operating theaters for emergency C-sections [27]. Investments in monitoring devices that can detect problems early will help the midwives in prioritizing the patients that need closer attention or emergency care. Operating theaters for C-section will reduce unnecessary delays in treatment, and thus avoiding preventable deaths.

Being mistreated by their patients and having a bad reputation in society are also factors that were reported to be challenging for the midwives, contributing to the aforementioned feeling of demoralization. Women giving birth in healthcare facilities in Tanzania report limited support, neglect and physical and verbal abuse during labor [17]. A possible way to alter the midwives’ bad reputation is tackling some of the other challenges they face. If the midwives were more motivated, they might be more polite and attentive towards the patients, something that may lead to more frequent follow ups and better care, according to Franco et al. [20]. Increasing the amount of staff would be helpful since this will give the midwives more time to care for each patient. The same is true for obtaining the necessary equipment and facilities, as these are integral factors in providing quality healthcare [24].

The population’s knowledge of reproductive health was reported to be limited and increasing this can be another way to aid the midwives’ reputation and position in society. The knowledge will help patients understand the importance of getting qualified assistance during labor and the effect this has for birth outcome. Increased knowledge in itself has been documented to increase rates of facility-assisted deliveries, which in turn will have a positive effect on both mother and child mortality [28]. Higher levels of education have been shown to increase the use of delivery services [29]. Focusing on a general increase in education in the region, with emphasis on awareness of danger signs during pregnancy and delivery [30], might therefore have an effect on birth outcome, and help eliminate the problem of seeking professional help too late.

## Strengths and limitations

In this study we have described what kind of challenges midwives meet practicing midwifery in a busy Tanzanian Referral Hospital, overcrowded with women in the wards and delivery rooms. One of the main strengths of the study is that it offers the midwives’ own perspectives on their working situation and their own thoughts about possible measures to improve it. This can create a foundation for further research on interventions that are adapted to the local context. A second strength is using a Tanzanian midwife as a moderator and translator. She understood the midwives’ situation, thereby making the participants feel more comfortable and willing to share their stories. Moreover, the preliminary results from the FGDs were presented both to the staff and administration at the hospital. This made it possible for the midwives to confirm the findings, which strengthens their credibility, and made sure that the midwives’ concerns were heard on an organizational level.

However, focusing solely on the perspective of the midwives is a limitation. Including patients, doctors or the hospital administration in the study would provide more diverse experiences and broader insight into the situation at the hospital. Other topics that affect the perceptions of workload and women’s utilization of the provided health care, such as education, culture, religion, economic status, women’s health seeking behavior and knowledge of danger signs, could also have been discussed further. A second limitation is that the study was conducted by two foreign researchers without the ability to speak Kiswahili and insight into the local context of midwifery. This might have affected the interpretation of the material. Using English was considered, but the authors concluded that this would lead to a more substantial loss of information than a translation process would. Measures taken to limit these disadvantages were spending time in the wards to familiarize with the midwives and their working environment before the interviews, having a co-author and local supervisor who works at the hospital and knows the midwives well, and using a moderator and translator who is familiar with the local context of midwifery, being a midwife herself. The local supervisor and co-author, who is fluent in Kiswahili, double-checked the recordings against the transcripts to make sure the quality of the translations was satisfactory, to limit the disadvantage of using only one translator.

## Trustworthiness

The credibility of this study was ensured by describing the qualitative research method, the use of FGDs, analyzing the data with STC, frequently revisiting the data, and presenting the content with quotes from the research participants. Furthermore, the collaboration of students and international researchers was important for gaining an in-depth understanding of the data. This collaboration included two Norwegian fifth year medical students, a Tanzanian supervisor who works as an obstetrician in a busy District Hospital, and a Scandinavian professor and obstetrician, familiar with the Tanzanian context and with vast experience of research in reproductive health in several other low-income settings. In addition, the FGDs were conducted, within a short period of time, by a Tanzanian midwife fluent in Kiswahili and with understanding of the context, allowing for consistency throughout the data collection process, and thereby increasing its dependability. All material was immediately translated to English after each FGD. A clear and detailed description of the study context and setting, as well as descriptions of the participants, the data collection and analysis processes has been provided for the readers to improve transferability.

## Conclusion

This study aims to show that midwives working at a regional referral hospital in Dar es Salaam face considerable challenges, both pertaining to the management of the healthcare service (locally and nationally), possibilities for advancement (education and promotion), availability of resources (materials and personnel) and reputation and knowledge in the population. The challenges they face, constitute barriers to providing good midwifery care for their patients, causes problems for them on a personal level, and demotivates them in their work. They therefore need to be addressed, both by government funders, health policy makers and regulators. A feeling of demoralization is particularly apparent. The main cause of this feeling is an absence of support and understanding from their leaders. Creating an arena for dialogue and implementing a more supportive leadership style would be efficient measures that can, and should, be taken to improve the midwives’ working conditions. Other important measures are reduction of workload, either with increased amount of staff or reorganization of the referral system, providing sufficient equipment, either through a larger supply or modification of polices, and increasing the knowledge level, both through providing training for the midwives and through educating the population. Taking these measures will improve the quality of care the midwives provide, which in turn can lead to improved health for both mothers and children in Tanzania.

## Paper context

Tanzania has a high fertility rate and lacks skilled health workers to provide care for delivering women. Combined, these factors cause excessive work-pressure for midwives in the country. Our research gives insight into midwives’ perception of their working situation and which measures they find necessary to implement to ease the burden. Findings from this study may provide a starting point for interventions to improve midwives’ working conditions and strengthen maternal and child health in Tanzania.
